# Rare association of peripapillary retinochoroidoscleral rupture and retrobulbar cyst: Clinical, imaging, and surgical insights

**DOI:** 10.1016/j.ajoc.2026.102597

**Published:** 2026-05-07

**Authors:** Yasaman Sadeghi, Esmaeil Asadi Khameneh, Arash Mirzaei, Mohammad Taher Rajabi, Amirhossein Aghajani, Nader Mohammadi, Seyed Mohsen Rafizadeh

**Affiliations:** Eye Research Center, Farabi Eye Hospital, Tehran University of Medical Sciences, Tehran, Iran

**Keywords:** Orbital reconstruction, Orbital trauma, Ocular trauma, Peripapillary rupture, Retrobulbar cyst, Scleral repair

## Abstract

**Purpose:**

A 17-year-old male presented with persistent visual loss and extensive orbital wall fractures following severe head and orbital trauma sustained in a motor vehicle accident.

**Observations:**

He was found to have a full-thickness peripapillary retinochoroidoscleral rupture associated with a retrobulbar cyst, possibly related to post-traumatic communication through the retinochoroidoscleral defect. Management consisted of barrier laser photocoagulation, lateral orbitotomy with cyst drainage, and primary scleral repair. Postoperatively, the patient developed a submacular hemorrhage, which was managed with intravitreal gas injection. Subsequently, orbital reconstruction with a Medpor wedge implant was performed to correct enophthalmos.

**Conclusions and importance:**

This rare case underscores the unusual association of traumatic globe rupture with retrobulbar cyst formation and highlights the surgical challenges as well as the long-term functional and cosmetic outcomes.

## 1 Introduction

Orbital trauma from high-energy accidents often results in orbital wall fractures, ocular motility restriction, and optic neuropathy. However, posterior globe rupture involving retinal, choroidal, and scleral laceration with secondary retrobulbar cyst formation is exceedingly uncommon. Orbital cysts typically arise from congenital, inflammatory, or iatrogenic causes, while post-traumatic retrobulbar cysts are rare and usually associated with severe ocular wall disruption.^1^^,^^2^ Traumatic retinal breaks most often occur in the peripheral retina; peripapillary or macular full-thickness defects are infrequently reported.^3^ Globe rupture secondary to blunt ocular trauma usually occurs at the thinnest areas of the scleral wall, commonly at or just posterior to the extraocular rectus muscle insertions or at the limbus.^4^ In contrast, isolated globe rupture in the peripapillary region, where the sclera is thickest, is exceptionally rare. When accompanied by orbital fractures, management requires a multidisciplinary surgical approach.

We report a rare case of a young male who developed a peripapillary full-thickness retinochoroidoscleral defect with probable retinal incarceration with a retrobulbar cyst following orbital trauma, and we describe the surgical management, complications, and long-term outcomes.

## 2 Case report

A 17-year-old male presented to our hospital with a history of head and orbital trauma sustained in a motor vehicle accident three months earlier. He had remained comatose for approximately one month following the injury. Upon regaining consciousness, he reported fluctuating vision loss in the right eye and ocular deviation. Informed consent was obtained for the use of clinical data and multimodal imaging obtained throughout the treatment timeline.

**Fig. 1:**
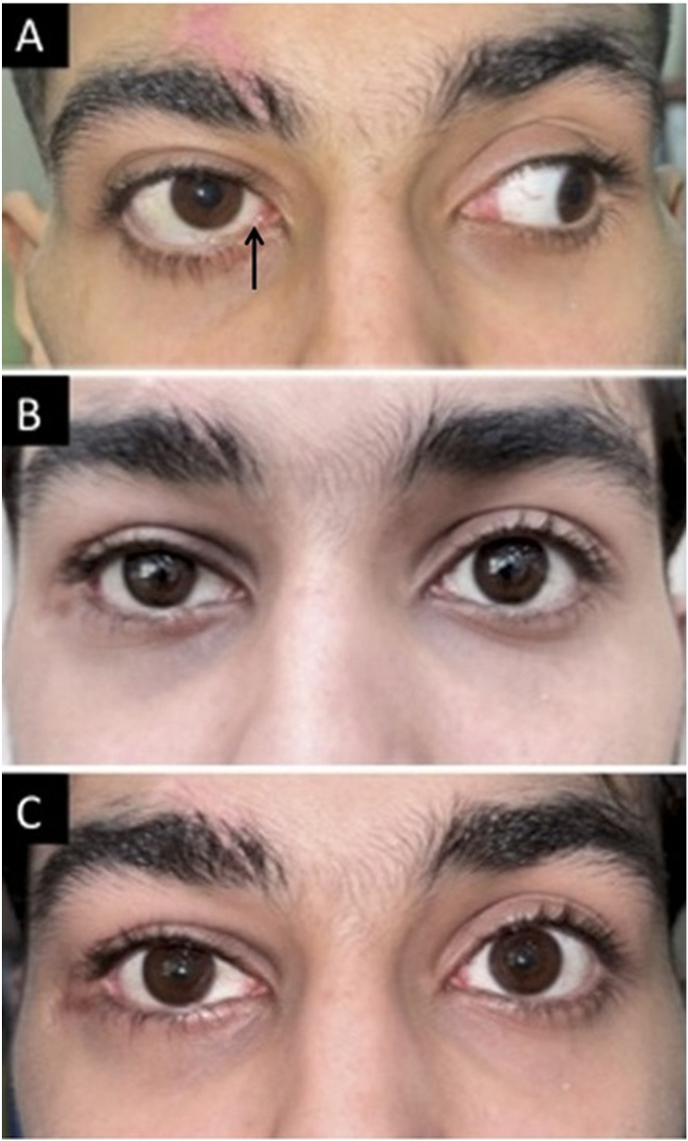
(A) Facial photograph at presentation showing limitation of adduction in the right eye with proptosis. black arrow indicates nasal scleral show adjacent to the limbus, consistent with an adduction deficit (B) After surgical removal of the orbital cyst, enophthalmos became evident. (C) Following placement of a Medpor wedge implant on the orbital floor, the enophthalmos was significantly reduced.

**Fig. 2:**
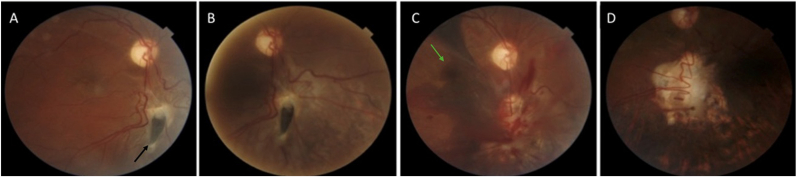
(A) Fundus photograph at presentation demonstrating a full-thickness retinochoroidoscleral defect with shallow subretinal fluid (black arrow). (B) One week after barrier laser treatment, the subretinal fluid had largely resolved, with visible scar formation at the laser treatment sites. (C) After surgical removal of the orbital cyst and primary repair of the retinochoroidoscleral laceration, a submacular hemorrhage was detected (green arrow), adjacent to the repaired retinochoroidoscleral defect. (D) Two months after intravitreal SF_6_ injection, the submacular hemorrhage had completely resolved, the retina was reattached, and the barrier laser scars were clearly visible. (For interpretation of the references to colour in this figure legend, the reader is referred to the Web version of this article.)

On examination, the patient was alert and oriented. Best-corrected visual acuity (BCVA) was counting fingers at 50 cm in the right eye and 20/20 in the left. A relative afferent pupillary defect (RAPD) was present in the right eye, consistent with traumatic optic neuropathy. External examination revealed right-sided 6 mm proptosis (Hertel: 28 mm OD vs. 22 mm OS) with hypotropia, exotropia, and restricted motility in adduction, elevation, and depression, consistent with pupillary-sparing third nerve palsy (Fig. 1A). Intraocular pressures were 10 mmHg OD and 16 mmHg OS. The anterior segment was unremarkable bilaterally, with no evidence of penetrating ocular trauma. Fundus examination of the right eye showed a peripapillary full-thickness retinochoroidoscleral defect with localized subretinal fluid, suspicious for retinal incarceration at the site of posterior scleral rupture rather than a typical rhegmatogenous break and mild optic nerve pallor (Fig. 2A). The left fundus was normal.

**Fig. 3:**
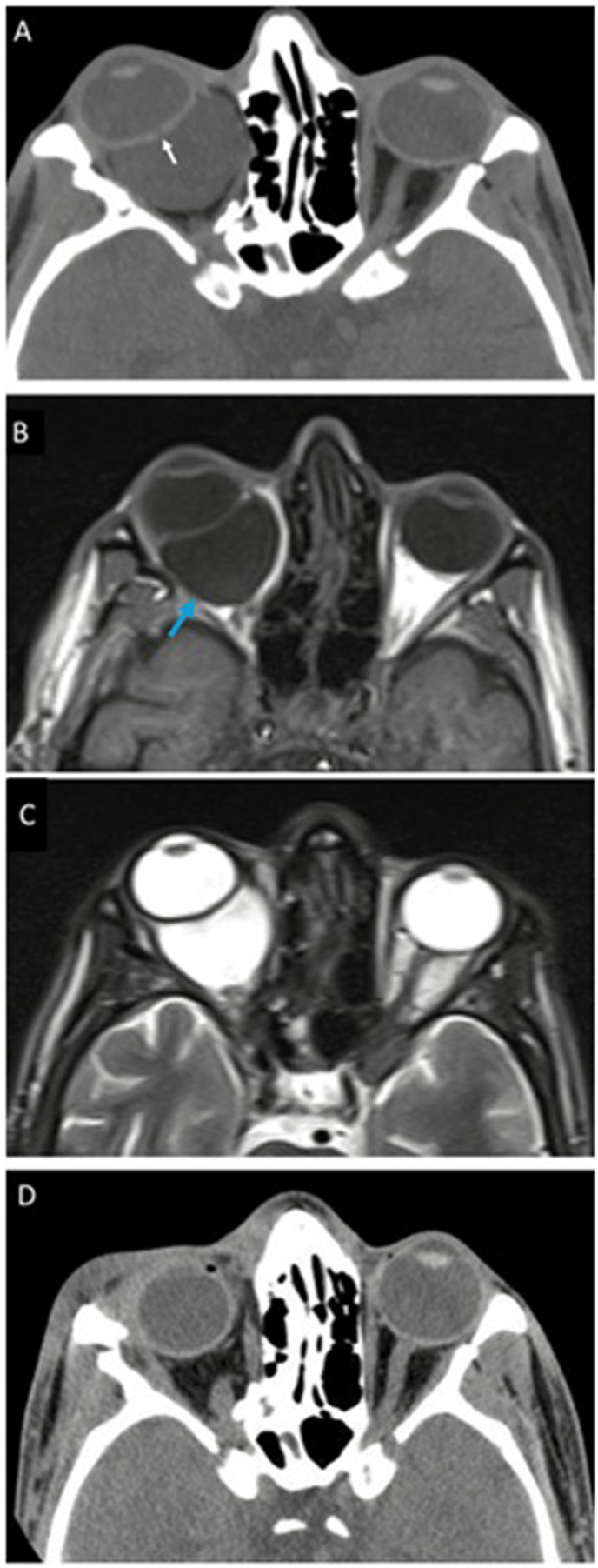
(A) Axial orbital computed tomography (CT) showing proptosis of the right eye with fractures of the lateral and medial orbital walls, and a retrobulbar cyst and the full-thickness retinochoroidoscleral defect (white arrow). (B) Magnetic resonance imaging (MRI) demonstrates a well-defined retrobulbar cystic lesion posterior to the globe, which was hypointense on T1-weighted sequences without contrast enhancement (blue arrow) and (C) hyperintense on T2-weighted images. (D) CT performed 4 weeks after surgical removal of orbital cyst. (For interpretation of the references to colour in this figure legend, the reader is referred to the Web version of this article.)

Orbital computed tomography (CT) demonstrated right-sided proptosis, fractures of all orbital walls, and a retrobulbar cyst (Fig. 3A). Magnetic resonance imaging (MRI) with and without gadolinium contrast was performed for further characterization of the cyst, revealing a well-defined, retrobulbar cystic lesion posterior to the globe, hypointense on T1-weighted sequences and hyperintense on T2-weighted images, consistent with a fluid-filled cyst (Fig. 3B and C). The lesion displaced the globe anteriorly and correlated with the site of scleral discontinuity near the optic disc.

**Fig. 4:**
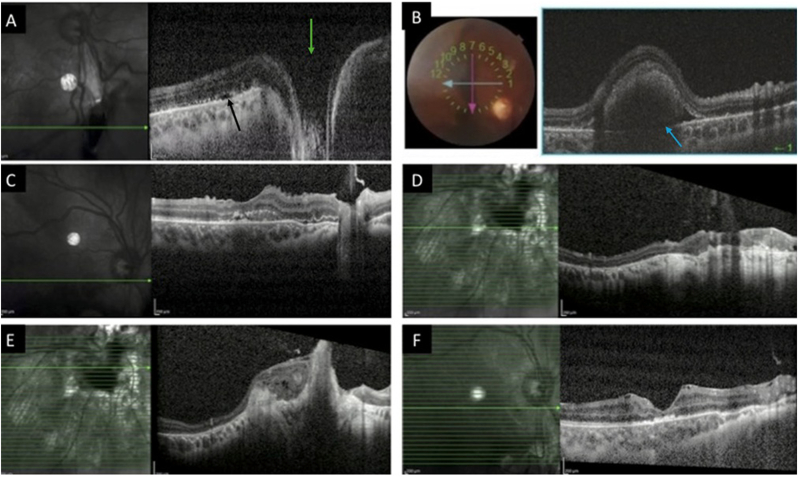
(A) Enhanced-depth imaging optical coherence tomography (EDI-OCT) one week after presentation demonstrates a full-thickness retinochoroidoscleral defect inferior to the optic disc (green arrow) with shallow subretinal fluid (black arrow). (B) After surgical removal of the orbital cyst and primary repair of the retinochoroidoscleral laceration, OCT revealed submacular hemorrhage (blue arrow). (C) Two weeks after intravitreal SF_6_ injection, EDI-OCT showed significant resolution of the submacular hemorrhage and (D,E) complete closure of the full-thickness break. (F) On EDI-OCT obtained two months after SF_6_ injection, restoration of the hyperreflective outer retinal bands was evident. (For interpretation of the references to colour in this figure legend, the reader is referred to the Web version of this article.)

At follow-up one week later, clinically evident subretinal fluid had largely resolved at follow-up. However, EDI-OCT demonstrated a shallow hyporeflective space adjacent to the defect, which may have represented residual structural alteration of the outer retina rather than active subretinal fluid accumulation (Fig. 4A). Barrier laser photocoagulation was applied circumferentially around the break, extending up to 500 μm from the optic disc, to reduce the risk of recurrent fluid accumulation or retinal detachment. One week later, the subretinal fluid had resolved, though the retinochoroidoscleral defect with probable retinal incarceration remained visible (Fig. 2B).

Surgical intervention was undertaken to drain the cyst. Under general anesthesia, a lateral orbitotomy via a lateral crease incision with bone removal was performed. Inferior transconjunctival orbitotomy and lateral canthotomy/cantholysis provided exposure. The cyst was dissected from surrounding tissues, except at its attachment to the globe. Controlled inflow through a limbal stab incision was used to maintain intraocular pressure. The cyst was punctured, drained of serous fluid, and opened, revealing fibrotic inner lining and apparent communication with the globe at the site of scleral disruption. The rupture site was repaired with 8-0 nylon interrupted sutures. Integrity of the closure was confirmed with high inflow testing. The orbital rim was repositioned, periosteum and canthal tendon reattached, and conjunctival and skin closure completed. The cyst wall was sent for histopathologic analysis, which revealed predominantly fibrotic tissue containing areas of hemorrhage, without evidence of a distinct epithelial lining. High-magnification H&E staining demonstrated dense fibrocollagenous stroma with blood breakdown products, consistent with a post-traumatic cystic process rather than a true epithelial cyst. Postoperatively, the patient received topical and systemic antibiotics and corticosteroids for 10 days.

One week postoperatively, a submacular hemorrhage detected in the right eye, likely tracking through the repaired peripapillary break into the subretinal space (Fig. 2, Fig. 4B). This was managed with intravitreal injection of 0.3 mL undiluted SF_6_ gas via pars plana following anterior chamber paracentesis. Given the patient's prior intracranial hemorrhage, intravitreal tissue plasminogen activator (tPA) and anti-VEGF therapy were avoided.

At two weeks, the subretinal hemorrhage had significantly reduced (Fig. 4C) and the retina was attached, with visible barrier laser scars. The full-thickness retinochoroidoscleral laceration site was successfully closed (Fig. 4D and E). At two months, the hemorrhage had resolved, the retina remained attached, and BCVA had improved to 20/200 (Fig. 2, Fig. 4F).

Five months later, persistent 5 mm enophthalmos (Hertel: 17 mm OD vs. 22 mm OS) prompted secondary orbital reconstruction (Fig. 1B). Via repeat inferior transconjunctival orbitotomy with lateral canthotomy/cantholysis, adhesions were released and a large Medpor wedge implant was placed on the orbital floor. Closure included periosteal suturing, conjunctival repair, lateral canthopexy, and temporary Frost suture fixation. Postoperative management again included systemic and topical antibiotics and corticosteroids. At final follow-up, Hertel measurements improved to 21 mm OD versus 22 mm OS, with a stable globe and no recurrence of the cyst. BCVA remained stable at 20/200 (Fig. 1C). Gradual resolution of third nerve palsy was seen over time, with a small degree of residual paresis remaining on last examination.

## 3 Discussion

Orbital trauma can lead to multiple sequelae, including fractures, motility restriction, optic nerve injury, and, more rarely, secondary cyst formation. Retrobulbar cysts are uncommon and typically arise from congenital choristomas, inflammatory processes, or surgical complications; however, post-traumatic cysts have also been reported.^1^^,^^5^ In the present case, several mechanisms may explain the development of the retrobulbar cyst in this case. One possibility is limited extrusion of intraocular fluid or vitreous material through the full-thickness retinochoroidoscleral defect. However, given the patient's young age and absence of posterior vitreous detachment, sustained vitreous egress is less likely. Alternatively, the cyst may represent a post-traumatic reactive serous collection, an organized hematic cyst, or fibrocollagenous pseudocyst formation adjacent to the site of scleral disruption. Histopathologic findings demonstrating fibrotic tissue without epithelial lining support a post-traumatic reactive process rather than a true epithelial cyst.

Traumatic retinal breaks most often occur in the peripheral retina, with peripapillary tears being distinctly uncommon.^3^^,^^6^ Full-thickness ruptures involving the sclera and choroid are even more unusual, particularly in regions where the sclera is thickest, such as near the optic nerve head. Their association with orbital cysts has rarely been documented. EDI-OCT was instrumental in confirming the depth of the rupture and in guiding surgical planning.

Management was complex and required a staged, multidisciplinary approach. Barrier laser photocoagulation around the full-thickness retinochoroidoscleral defect was initially effective in limiting subretinal fluid; however, the persistent communication with the retrobulbar cyst necessitated orbitotomy and direct closure of the defect. Without closure, persistent communication across the defect could theoretically have compromised the laser barrier and predisposed to recurrent fluid accumulation. Similar strategies have been described in cases of traumatic scleral rupture with intraorbital extension.^7^

The development of submacular hemorrhage following cyst removal and scleral repair may have been multifactorial. Potential mechanisms include transient intraoperative pressure fluctuations, delayed bleeding from previously contused choroidal vasculature, or iatrogenic microvascular trauma during manipulation. Although traumatic choroidal neovascularization is a theoretical consideration in delayed post-traumatic hemorrhage, serial OCT imaging did not demonstrate features suggestive of CNV in this case. This was successfully managed with intravitreal gas tamponade, while intravitreal tPA or anti-VEGF was avoided given the patient's neurological history. Subsequently, enophthalmos from orbital wall fractures required secondary orbital reconstruction with a Medpor implant, which restored orbital volume and achieved a satisfactory cosmetic outcome, consistent with prior reports of post-traumatic orbital repair.^8^

Post-traumatic orbital repair is essential to restore orbital volume, correct enophthalmos, and re-establish ocular motility. Alloplastic materials such as porous polyethylene (Medpor) are widely used in orbital reconstruction because of their biocompatibility, stability, and low complication rates, providing favorable cosmetic and functional outcomes in patients with orbital floor fractures.^8^

To our knowledge, this represents the first reported case of posterior globe rupture near the optic nerve head associated with orbital cyst formation, successfully managed through sequential ophthalmic and oculoplastic procedures. This case highlights the value of multimodal imaging, careful surgical staging, and multidisciplinary collaboration in addressing rare and complex orbital trauma.

## CRediT authorship contribution statement

**Yasaman Sadeghi:** Writing – original draft, Data curation. **Esmaeil Asadi Khameneh:** Writing – review & editing, Visualization, Validation, Supervision. **Arash Mirzaei:** Writing – review & editing, Writing – original draft, Visualization, Supervision, Investigation. **Mohammad Taher Rajabi:** Visualization, Supervision, Conceptualization. **Amirhossein Aghajani:** Writing – review & editing, Visualization, Supervision. **Nader Mohammadi:** Writing – review & editing, Supervision, Conceptualization. **Seyed Mohsen Rafizadeh:** Writing – review & editing, Writing – original draft, Visualization, Supervision, Project administration, Methodology, Conceptualization.

## Patient consent

Written informed consent to publish this case report and associated images was obtained from the patient in writing.

## Claim of priority

After conducting a comprehensive literature review on using PubMed, Google Scholar, and Scopus with the keywords ‘peripapillary rupture,’ ‘retinochoroidoscleral rupture,’ ‘retrobulbar cyst,’ and ‘ocular trauma,’ we did not find any prior reports describing the association of a peripapillary full-thickness retino-choroidoscleral rupture with a retrobulbar cyst.

## Disclosures

The following authors have no financial disclosures: Y.S., E.A.K., A.M., M.T.R., A.A., N.M., S.M.R.

## Authorship

All authors attest that they meet the current ICMJE criteria for authorship.

## Funding

No funding or grant support.

## Declaration of competing interest

The authors declare that they have no known competing financial interests or personal relationships that could have appeared to influence the work reported in this paper.
